# Are there Disease-Specific Functional Features of Cardiac Mechanics?

**DOI:** 10.31083/j.rcm2504137

**Published:** 2024-04-07

**Authors:** Attila Nemes

**Affiliations:** ^1^Department of Medicine, Albert Szent-Györgyi Medical School, University of Szeged, H-6725 Szeged, Hungary

In recent decades, cardiovascular imaging diagnostics has evolved enormously. This development has made it possible to perform detailed non-invasive analyses that do not burden the patient. This technical development has taken place not only in the field of echocardiography, but in case of cardiac magnetic resonance imaging (cMRI) as well. The use of modern cardiovascular imaging diagnostics is now essential, as they enable accurate, validated, non-invasive and detailed analysis of the heart chambers. Accurate data regarding dimensions, volumes and functional characteristics of the cardiac cavities can be obtained, taking into account the changes that take place during the cardiac cycle. Moreover, ’function’ is a broad concept, since in addition to contractility-relaxation of the myocardial wall and its regions/segments, chamber-related volume-based parameters (such as the ejection fraction) can also be determined [1, 2].

Accordingly, we now have at our disposal scientific materials, that analyze the abnormalities of the heart chambers linked to certain pathologies in detail. The question may rightly arise as to whether these differences are disease-specific and whether they will be used later in the clinics as a diagnostic and prognostic tool. Although the literature data are promising, since it seems that certain pathologies may be accompanied by specific volumetric and functional abnormalities [3, 4, 5]. However, knowing this, their age and gender dependency cannot be excluded, and the duration of the disease and the accompanying factors causing it can also affect the parameters.

The quantitative characteristics of wall contractility (deformation), the so-called ‘strains’, can be calculated even in relation to the 3 directions of space (radial, circumferential, longitudinal). In addition, the left ventricle (LV) has a special movement similar to wringing out a towel, which is called LV twist [6]. Nowadays, in addition to echocardiography, even cMRI may be suitable for their characterization in clinical circumstances. The determination of these parameters is not only important because they may have diagnostic significance, but their prognostic significance may also be pronounced. cMRI can be suitable for tissue-specific characterization as well [2].

It is known from recently published reviews that characteristic abnormalities can be confirmed for the left (LA) and right atria (RA) and the LV in the presence of certain disorders [3, 4, 5]. Recent publications in the *Reviews in Cardiovascular Medicine* may strengthen this concept.

Amyloidosis is a systemic disease characterized by the accumulation of amyloid fibrils in various organs including the cardiac structures. The extent of organ involvement determines the degree of cardiac impairment, which can significantly impact the prognosis. cMRI-based analysis of cardiac amyloidosis was found to be an accurate method among others not only for differential diagnosis of LV hypertrophy phenotypes (tissue characterization), but also for monitoring disease progression and response to treatment [2].

The number of patients with congenital heart disease living into adulthood has increased significantly in recent decades due to the fact that both early detection, diagnostic and surgical techniques, and perioperative treatment have undergone significant improvement. Although there is a limited number of patients with single ventricle (SV), accurate assessment of their heart is essential. The situation is further complicated by the fact that, in addition to the small number of SV cases, there are several surgical procedures (Norwood, Fontan, etc.) used in the clinical practice. Nevertheless, strain measurements were found to provide useful additional information in both ventricular and atrial analyses [7].

New therapeutic procedures affecting the right heart required significant progress in its assessment. It was a problem that the right ventricle (RV) differs from the LV in its structure, shape and function as well. Nowadays, not only recent advanced echocardiographic techniques, but also cMRI is a suitable method for assessing RV. Moreover, prognostic impact of derived parameters has even been confirmed [8].

Similarly to the RV, the assessment of the atria was neglected in the past due to technical difficulties. Due to technological developments, it is now possible to analyze all phases of the atrial function in detail. Volumetric and strain analysis of the systolic reservoir, early diastolic conduit and late diastolic active contraction phases can be easily performed with recent cardiovascular imaging methods [9].

In addition to strain-based measurements, determination of the so-called myocardial work indices arose as a new diagnostic tool. For the ideal characterization of the LV function independent of load, the determination of pressure-volume relationships according to cardiac cycle would be suitable. For the characterization of regional myocardial work, a combination of LV pressure (which can be replaced by blood pressure readings on the upper arm) and strain parameters can be used to create such parameters like constructive, effective and wasted work of myocardium during the heart cycle [10]. This sort of approach is new with well-defined normal reference values [11].

There is a growing number of publications of study results analysing differences in certain diseases in several heart cavities (e.g. LV and LA) at the same time. Moreover, their combination and prognostic significance were also investigated [12].

The above findings not only highlight that the new imaging methods are suitable for detailed volumetric and functional LV analysis in certain (even rare) pathologies, but such studies can also be performed in relation to the RV and the atria as well. Combining them can help to find disease-specific abnormalities that may have diagnostic or even prognostic significance, but this may be the subject of future investigations.

## References

[1] Nemes A, Kalapos A, Domsik P, Forster T (2012). Three-dimensional speckle-tracking echocardiography – a further step in non-invasive three-dimensional cardiac imaging. *Orvosi Hetilap*.

[2] Tana M, Tana C, Panarese A, Panarese C, Ricci F, Porreca E (2023). Clinical and Cardiovascular Magnetic Resonance Imaging Features of Cardiac Amyloidosis. *Reviews in Cardiovascular Medicine*.

[3] Nemes A, Domsik P, Kalapos A, Forster T (2016). Is three-dimensional speckle-tracking echocardiography able to identify different patterns of left atrial dysfunction in selected disorders?: Short summary of the MAGYAR-Path Study. *International Journal of Cardiology*.

[4] Nemes A, Domsik P, Kalapos A, Kormányos Á, Ambrus N, Forster T (2018). Three-dimensional speckle-tracking echocardiography detects different patterns of right atrial dysfunction in selected disorders: a short summary from the MAGYAR-Path Study. *Quantitative Imaging in Medicine and Surgery*.

[5] Nemes A (2023). Left Ventricular Deformation and Rotational Mechanics in Various Pathologies-The Role of the Pattern of Abnormalities. *Journal of Clinical Medicine*.

[6] Nakatani S (2011). Left ventricular rotation and twist: why should we learn. *Journal of Cardiovascular Ultrasound*.

[7] Kleitsioti P, Koulaouzidis G, Giannakopoulou P, Charisopoulou D (2023). The Role of Myocardial Strain Imaging in the Pre- and Post-Operative Assessment of Patients with Single Ventricle. *Reviews in Cardiovascular Medicine*.

[8] Bo K, Zhou Z, Sun Z, Gao Y, Zhang H, Wang H (2022). Prognostic Value of Cardiac Magnetic Resonance in Assessing Right Ventricular Strain in Cardiovascular Disease: A Systematic Review and Meta-Analysis. *Reviews in Cardiovascular Medicine*.

[9] Marincheva G, Iakobishvili Z, Valdman A, Laish-Farkash A (2022). Left Atrial Strain: Clinical Use and Future Applications—A Focused Review Article. *Reviews in Cardiovascular Medicine*.

[10] Rabkin SW (2023). Assessing Myocardial Strain and Myocardial Work as a Marker for Hypertensive Heart Disease: A Meta-Analysis. *Reviews in Cardiovascular Medicine*.

[11] Manganaro R, Marchetta S, Dulgheru R, Ilardi F, Sugimoto T, Robinet S (2019). Echocardiographic reference ranges for normal non-invasive myocardial work indices: results from the EACVI NORRE study. *European Heart Journal Cardiovascular Imaging*.

[12] Liu S, Li Y, Lian J, Wang X, Li Y, Wang D (2023). Prognostic Significance of Biventricular and Biatrial Strain in Dilated Cardiomyopathy: Strain Analysis Derived from Cardiovascular Magnetic Resonance. *Reviews in Cardiovascular Medicine*.

